# Supplementary figures and images for: Splicing-aware scRNA-Seq resolution reveals execution-ready programs in effector Tregs
Source: PLoS Comput Biol. 2025 Nov 10;21(11):e1013682. doi: 10.1371/journal.pcbi.1013682 (PMC12646427; doi:10.1371/journal.pcbi.1013682)

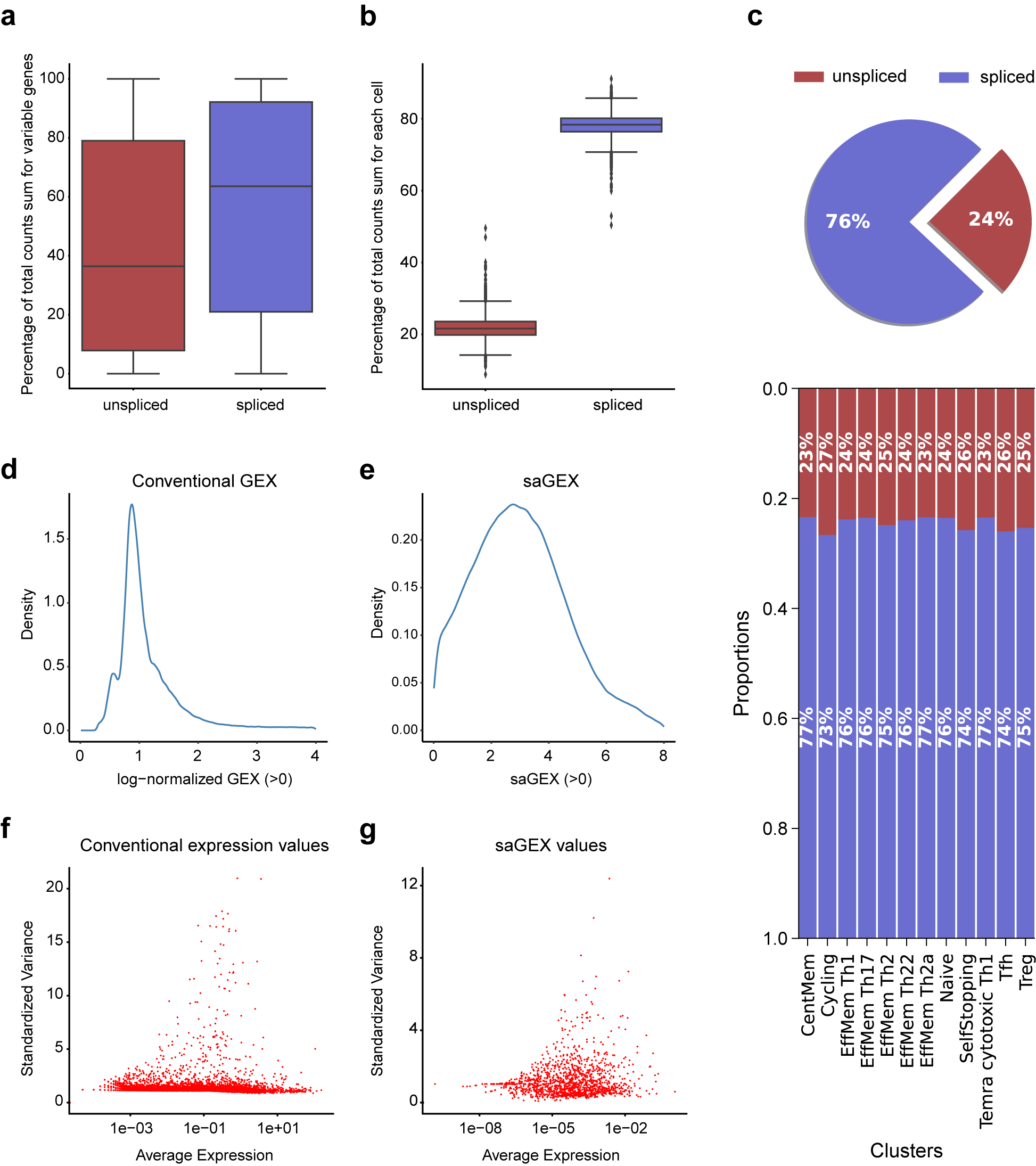

Supplement: S1 Fig — a,b,c. Relative proportion of spliced versus unspliced UMI per variable gene chosen for downstream analysis (a), per cell (b) and per scRNA-Seq cluster (c) as determined by velocyto. d,e,f,g. Comparison of value distribution between conventional expression and SANSARA-generated values. Density plots of log-normalized GEX and saGEX values (d,e). Mean-variance plot of log-normalized GEX and saGEX values as calculated by Seurat. Dots correspond to genes (f,g). (TIF) [file pcbi.1013682.s001.tif]

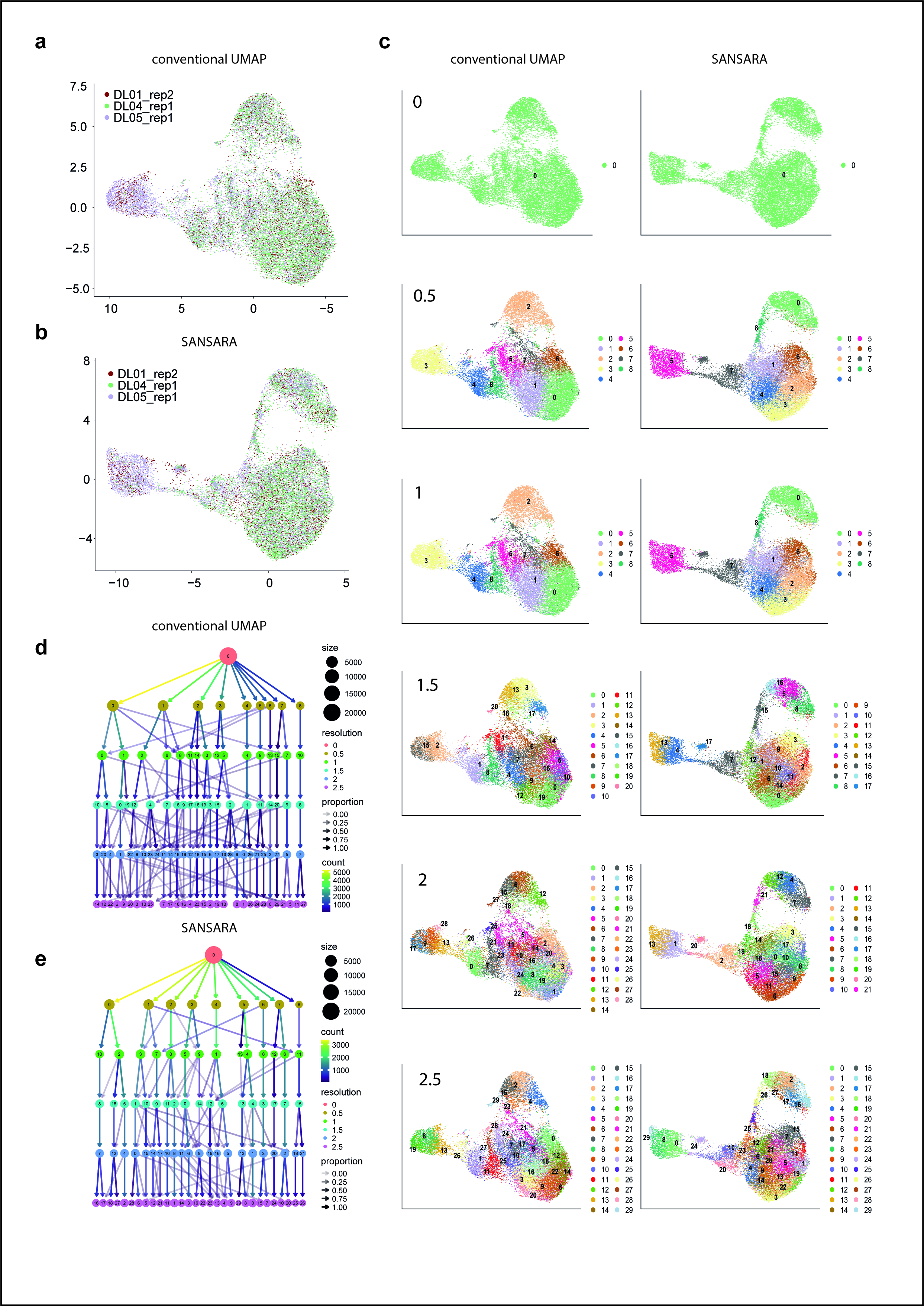

Supplement: S2 Fig — a,b. Harmony integration of scRNA-Seq data for the three donors performed with conventional (a) and splicing-aware (b) datasets. c. Clustering at different UMAP resolutions. d,e. Clustering trees for splicing-unaware (d) and splicing-aware (e) datasets. (TIF) [file pcbi.1013682.s002.tif]

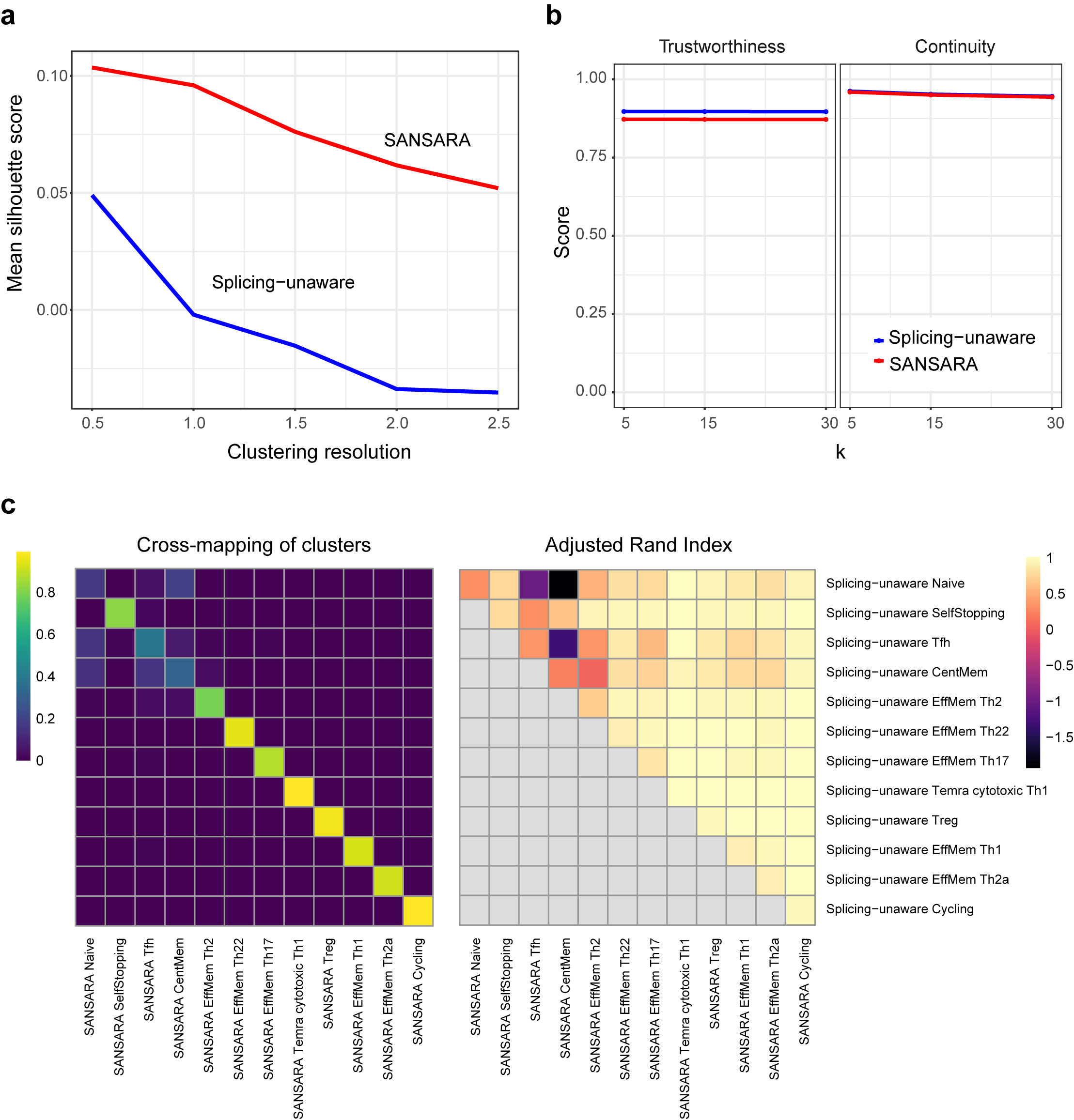

Supplement: S3 Fig — a. Comparison of silhouette scores on multiple resolutions between conventional splicing-unaware and SANSARA methods. Larger score points to greater separation of the clusters. b. Trustworthiness and continuity metrics at three k-values (5, 15, 30) for splicing-unaware and SANSARA dimensionality reduction step from PCA to UMAP. Values reflect the relative preservation of neighbors in UMAP compared to PCA. c. Left: Correspondence between clusters produced by splicing-unaware analysis and SANSARA. Each row identifies the cross-mapping of clusters from the different methods, normalized by the cluster abundance as calculated by Jaccard index of similarity. Right: Adjusted Rand Index. Pairwise heatmap shows which clusters of the reference (conventional splicing-unaware analysis) retain their integrity in SANSARA clustering. Higher index means the two clustering algorithms agree on which cells belong together and which are separated. (TIF) [file pcbi.1013682.s003.tif]

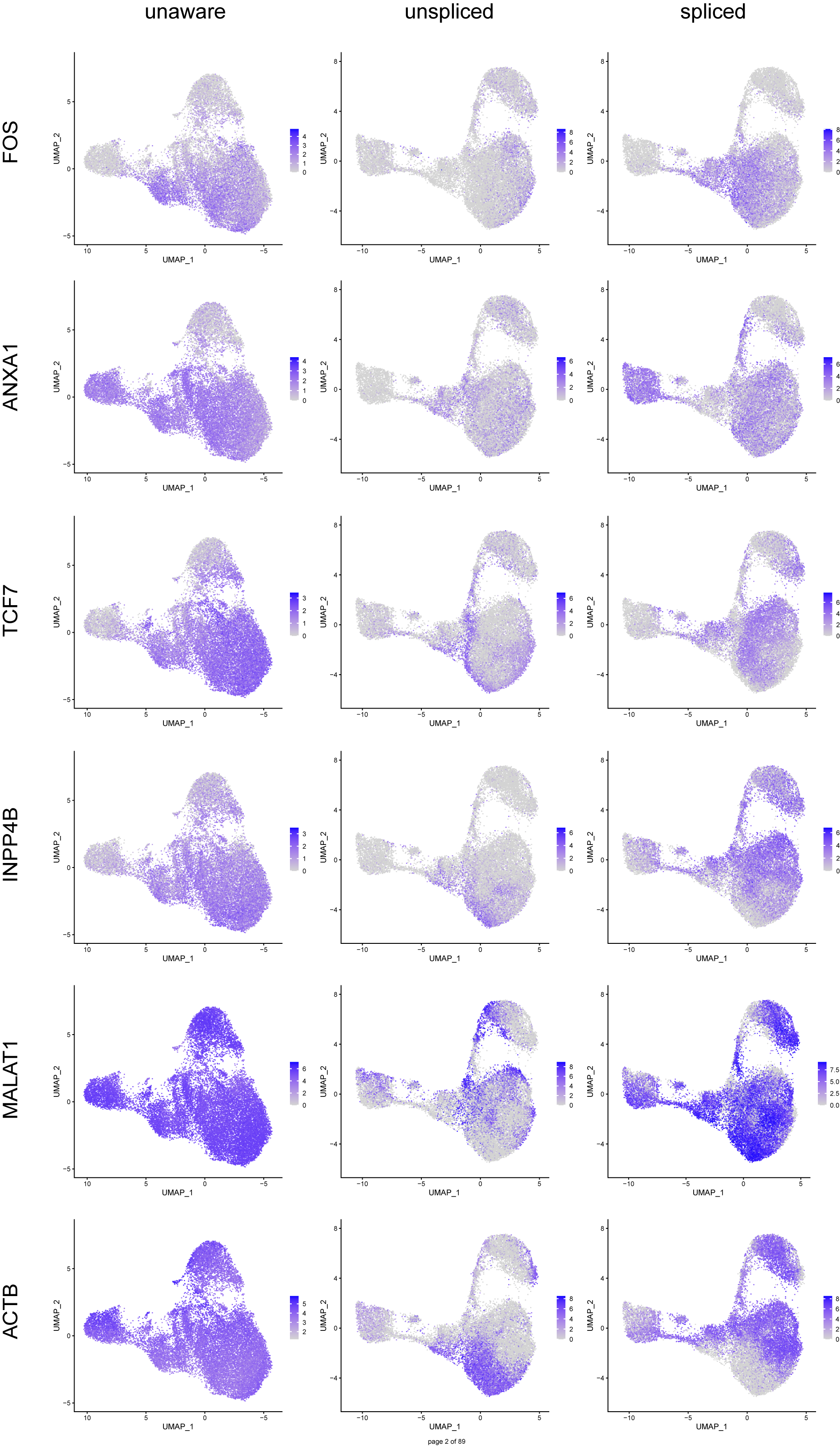

Supplement: S4 Fig — Splicing-unaware UMAP plots are shown at left; center and right panels show splicing-aware UMAP plots. FOS—encoding a c-Fos protein which interacts with c-Jun, forming heterodimeric AP-1 transcription factor that prominently affects CD4+ T cell differentiation [80]. ANXA1—encoding Annexin A1, the key driver of glucocorticoid anti-inflammatory effects, involved in T-cell differentiation, altering the strength of TCR signaling [81] and Th1-Th2 counterbalance driven by GATA3 and TBX21 transcription factors [82]. TCF7—encoding transcription factor T cell factor 1 which marks CD4 + T cells ability to self-renew [34] and which expression goes down along with effector T cell differentiation [83], especially towards CD4 + cytotoxic T cells [84]. INPP4B—encoding inositol poly-phosphate 4-phosphatase that was suggested to play role in T cell proliferation, survival and differentiation [85]. MALAT1—long noncoding RNA, reported as regulator of helper T cell differentiation from naïve CD4 + T cells [38]. ACTG1 and ACTB—cytoskeleton-related protein genes [32]. (TIF) [file pcbi.1013682.s004.tif]

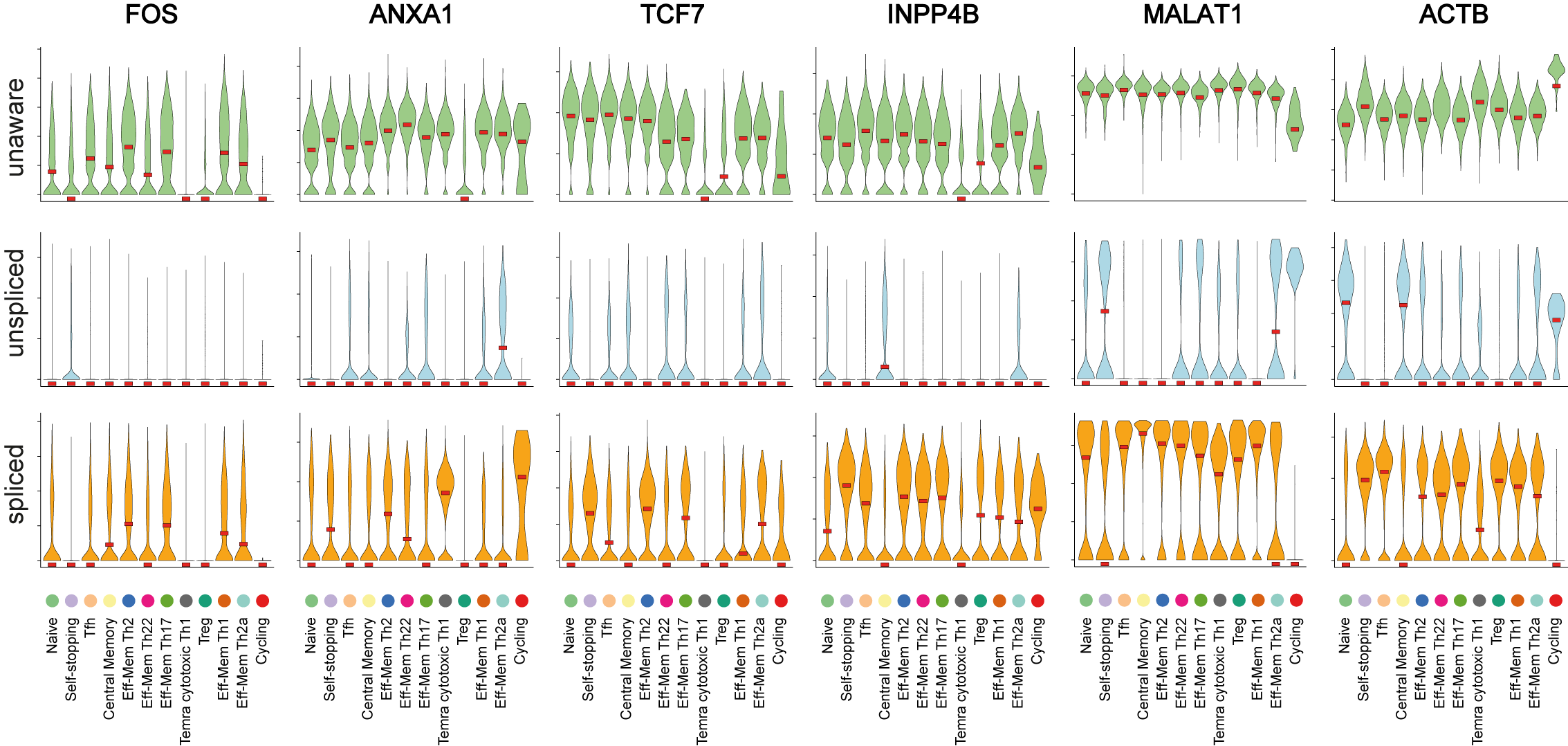

Supplement: S5 Fig — Violin plots of splicing-unaware (top) and -aware (middle, bottom) gene expression across clusters are shown. FOS—encoding a c-Fos protein which interacts with c-Jun, forming heterodimeric AP-1 transcription factor that prominently affects CD4+ T cell differentiation [80]. ANXA1—encoding Annexin A1, the key driver of glucocorticoid anti-inflammatory effects, involved in T-cell differentiation, altering the strength of TCR signaling [81] and Th1-Th2 counterbalance driven by GATA3 and TBX21 transcription factors [82]. TCF7—encoding transcription factor T cell factor 1 which marks CD4 + T cells ability to self-renew [34] and which expression goes down along with effector T cell differentiation [83], especially towards CD4 + cytotoxic T cells [84]. INPP4B—encoding inositol poly-phosphate 4-phosphatase that was suggested to play role in T cell proliferation, survival and differentiation [85]. MALAT1—long noncoding RNA, reported as regulator of helper T cell differentiation from naïve CD4 + T cells [38]. ACTG1 and ACTB—cytoskeleton-related protein genes [32]. (TIF) [file pcbi.1013682.s005.tif]

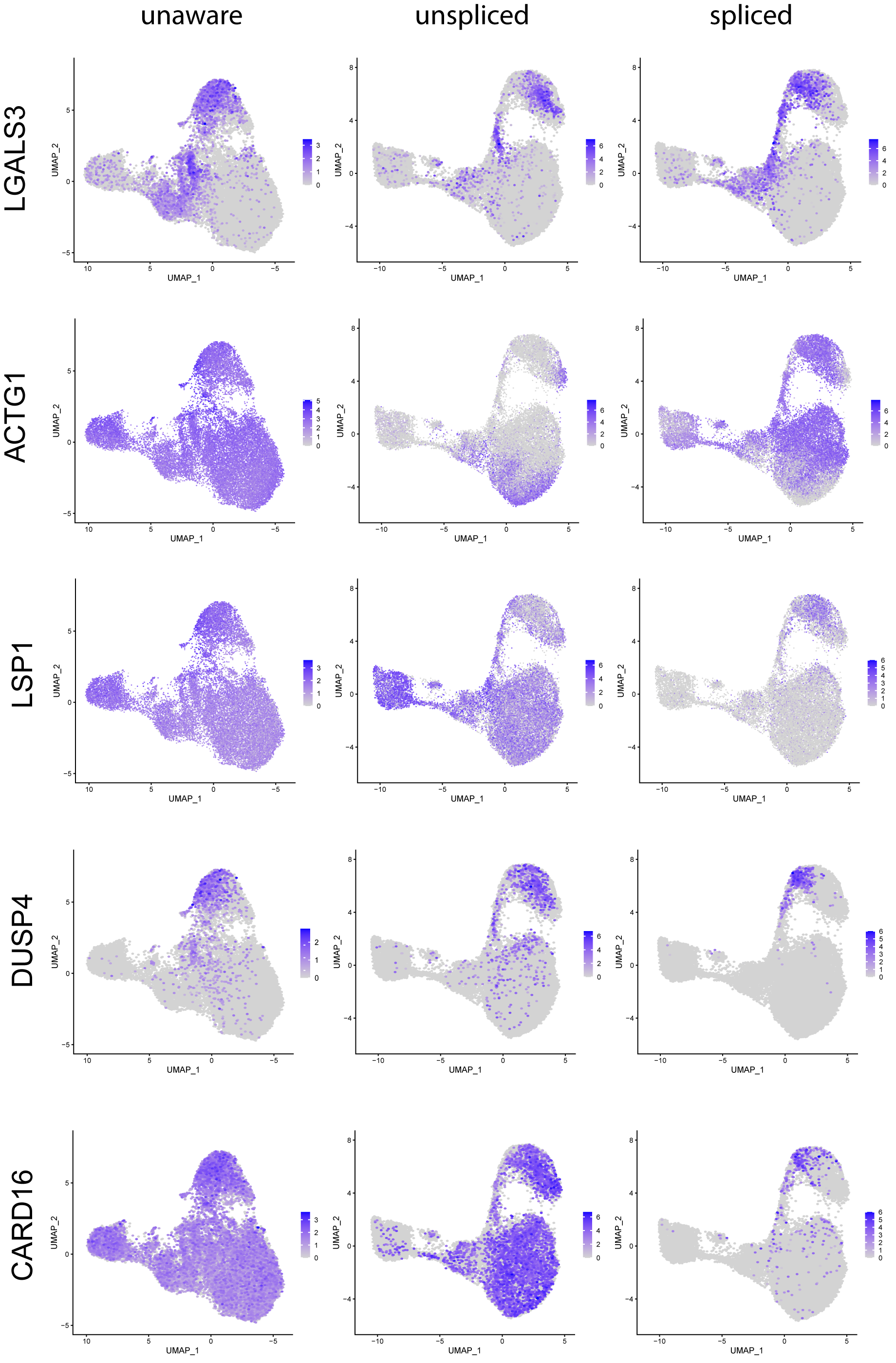

Supplement: S6 Fig — The lefthand column shows splicing-unaware UMAP plots, center and righthand columns show splicing-aware UMAP plots. (TIF) [file pcbi.1013682.s006.tif]
