# Supplementary material for: Splicing-aware scRNA-Seq resolution reveals execution-ready programs in effector Tregs
Source: PLoS Comput Biol. 2025 Nov 10;21(11):e1013682. doi: 10.1371/journal.pcbi.1013682 (PMC12646427; doi:10.1371/journal.pcbi.1013682)
Supplement: Attachment — Submitted filename: Reviewer_Comment.pdf [file pcbi.1013682.s012.pdf]

Comments on Splicing-aware scRNA-Seq resolution

The authors address the limitations of short-read sequencing technologies in scRNA-seq data analysis, particularly regarding splicing, which has often been treated in a post hoc manner. They propose a novel framework, SANSARA (Splicing-Aware scrNa-Seq AppRoAch), based on the insight that unspliced mRNA accumulation in the nucleus may reflect a transcription-independent reservoir characteristic of stable functional cell subsets. SANSARA computes a splicing-adjusted gene expression matrix (saGEX) by multiplying veloVI-derived splicing scores with normalized gene expression values (GEX) for each gene and cell. saGEX matrices from different donors are then integrated using the Harmony pipeline to correct for batch effects, followed by dimensionality reduction and clustering in Seurat.

The proposed framework is both methodologically innovative and biologically informative. However, I believe the manuscript would benefit from further clarification and enhancement in the following aspects:

1. Lack of quantitative validation: While SANSARA presents a conceptually compelling and biologically interpretable approach, the manuscript lacks rigorous quantitative comparisons to conventional splicing-unaware methods. For instance, incorporating metrics such as silhouette scores, cluster stability, or agreement with known or predicted cell type annotations (e.g., ARI or NMI) would help to demonstrate whether, and to what extent, the splicing-aware transformation enhances clustering performance. I acknowledge that such comparisons may be complicated by the use of predicted annotations—especially when derived from the same transformed data. Nonetheless, incorporating some form of independent or reference-based validation would help contextualize the added value of the proposed method.

2. Use of Harmony in the context of saGEX: Given that saGEX represents a novel transformation of scRNA-seq data incorporating splicing dynamics, the use of Harmony for donor integration is a reasonable—but nontrivial—choice. Since Harmony was originally developed for more conventional gene expression data, the authors should provide a brief discussion or cite relevant benchmarks to support the applicability of Harmony in this modified data context. Even a short acknowledgment of this methodological consideration would

reassure readers that this integration step was thoughtfully evaluated, given the novel nature of the input data.

3. Arbitrary choice of dimensionality: The analysis employs a fixed number of 25 principal components for dimensionality reduction, but this choice is not explicitly justified. Although it may work well in practice, it would be more rigorous to adopt a data-driven strategy—such as selecting the number of PCs based on explained variance (e.g., using elbow plots or cumulative variance thresholds). Even if the final results remain largely consistent, incorporating such criteria would improve methodological transparency and promote reproducibility.
