# Supplementary material for: Splicing-aware scRNA-Seq resolution reveals execution-ready programs in effector Tregs
Source: PLoS Comput Biol. 2025 Nov 10;21(11):e1013682. doi: 10.1371/journal.pcbi.1013682 (PMC12646427; doi:10.1371/journal.pcbi.1013682)
Supplement: Attachment — Submitted filename: Response to reviewers.pdf [file pcbi.1013682.s015.pdf]

We are grateful to all reviewers for the thorough work on our manuscript and the positive view. Please find our point-by-point response below.

Reviewer 1

Comments on Splicing-aware scRNA-Seq resolution.

The authors address the limitations of short-read sequencing technologies in scRNA-seq data analysis, particularly regarding splicing, which has often been treated in a post hoc manner.

They propose a novel framework, SANSARA (Splicing-Aware scrNa-Seq AppRoAch), based on the insight that unspliced mRNA accumulation in the nucleus may reflect a transcription-independent reservoir characteristic of stable functional cell subsets. SANSARA computes a splicing-adjusted gene expression matrix (saGEX) by multiplying veloVI-derived splicing scores with normalized gene expression values (GEX) for each gene and cell. saGEX matrices from different donors are then integrated using the Harmony pipeline to correct for batch effects, followed by dimensionality reduction and clustering in Seurat.

The proposed framework is both methodologically innovative and biologically informative.

Thank you for your deep understanding and positive view.

However, I believe the manuscript would benefit from further clarification and enhancement in the following aspects:

1. Lack of quantitative validation: While SANSARA presents a conceptually compelling and biologically interpretable approach, the manuscript lacks rigorous quantitative comparisons to conventional splicing-unaware methods. For instance, incorporating metrics such as silhouette scores, cluster stability, or agreement with known or predicted cell type annotations (e.g., ARI or NMI) would help to demonstrate whether, and to what extent, the splicing-aware transformation enhances clustering performance. I acknowledge that such comparisons may be complicated by the use of predicted annotations—especially when derived from the same transformed data. Nonetheless, incorporating some form of independent or reference-based validation would help contextualize the added value of the proposed method.

Thank you for the rational point. We added the following analysis to the **Results** section, along with a new **Supplementary Figure 3**:

“We used several metrics to evaluate clustering performance of splicing-aware dimensionality reduction with the SANSARA approach. First, we calculated Silhouette scores on multiple resolutions and compared between splicing unaware and splicing aware datasets. For each resolution value, we computed the average silhouette score across all resulting clusters. This analysis showed that SANSARA outperforms conventional analysis on most resolutions (**Supplementary Fig. 3a**).

Next, we compared how faithfully a low-dimensional embedding (UMAP) preserves the neighborhood structure of a higher-dimensional reference space (Harmony-corrected PCA) across methods, as SANSARA values are different from conventional expression values. Trustworthiness is a metric that goes down if UMAP invents spurious neighbors (cells that weren’t close in PCA space), and continuity assesses the loss of true neighbors in UMAP compared to PCA. Both scores are in (0, 1) range and

are calculated at several k values (the neighborhood size). The results show that SANSARA analysis consistently preserves the PCA neighborhood structure at comparable level to the conventional splicing-unaware analysis (**Supplementary Fig. 3b**).

We also assessed the correspondence between clusters produced by splicing-unaware analysis and SANSARA at the same resolution (**Supplementary Fig. 3c**) and adjusted Rand Index (ARI), which measures the proportion of cell pairs that remain in the same clusters across methods while accounting for cluster sizes (**Supplementary Fig. 3d**). Both analyses showed that most of the clusters are nearly identical between the methods, with the exception of Naive, Tfh and CentMem, which are traditionally challenging to define.

Based on these results, we conclude that integration using single-cell transcriptome data with splicing taken into account is comparable to conventional scRNA-Seq data integration. Both approaches worked similarly, even though the splicing-aware dataset contains fundamentally different information."

2. Use of Harmony in the context of saGEX: Given that saGEX represents a novel transformation of scRNA-seq data incorporating splicing dynamics, the use of Harmony for donor integration is a reasonable—but nontrivial—choice. Since Harmony was originally developed for more conventional gene expression data, the authors should provide a brief discussion or cite relevant benchmarks to support the applicability of Harmony in this modified data context. Even a short acknowledgment of this methodological consideration would reassure readers that this integration step was thoughtfully evaluated, given the novel nature of the input data.

Thank you for the suggestion. We added the following sentence to the Results section, along with a new **Supplementary Figure 1**:

"Harmony algorithm was chosen because it operates on the level of low-dimensional PCA embedding and does not require raw counts with negative binomial assumption, as PCA uses scaled and centered data. The distribution and mean-variance relationship of non-zero saGEX expression values (as calculated by Seurat) were generally preserved relative to the conventional analysis, supporting the applicability of Harmony (**Supplementary Figure 1d-g**)."

However, saGEX matrix is more zero-inflated due to the process of SANSARA (data not shown).

To confirm that Harmony is indeed applicable and works as intended we calculated and compared Local Inverse Sympson Index (LISI) scores of PCA before and after correction, showing that Harmony increased batch mixing (integration Local Inverse Sympson Index, iLISI) while preserving cell type composition (cell type Local Inverse Sympson Index, cLISI) (**Response Figure 1**).

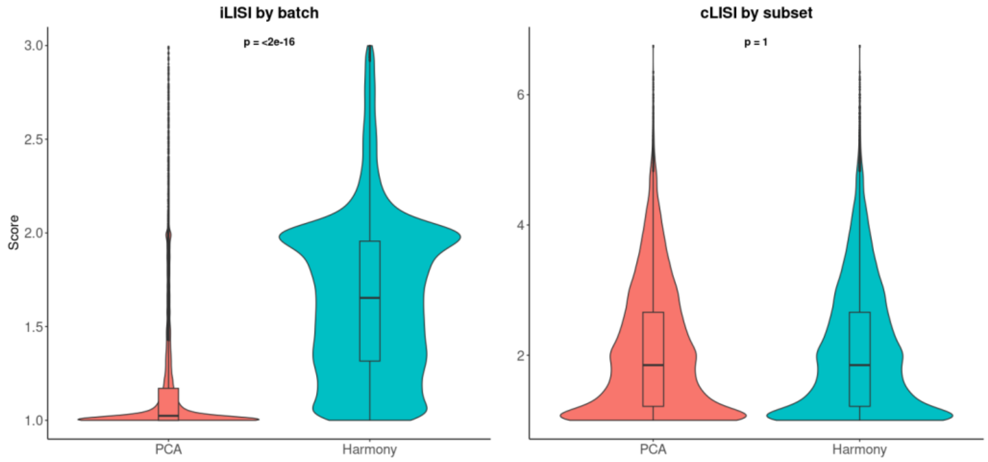

Response Figure 1. LISI scores for PCA space of SANSARA dataset before and after Harmony correction. Higher iLISI indicates better batch mixing, and no changes in cLISI shows preserved cell-type structure in the PCA neighborhoods. Wilcoxon test p-values are shown.

Additionally, lack of difference for Trustworthiness and Continuity metrics of UMAP/Harmony embeddings between SANSARA and traditional expression also suggests that Harmony is suitable for saGEX values integration (**Supplementary Figure 3b**).

3. Arbitrary choice of dimensionality: The analysis employs a fixed number of 25 principal components for dimensionality reduction, but this choice is not explicitly justified. Although it may work well in practice, it would be more rigorous to adopt a data-driven strategy—such as selecting the number of PCs based on explained variance (e.g., using elbow plots or cumulative variance thresholds). Even if the final results remain largely consistent, incorporating such criteria would improve methodological transparency and promote reproducibility.

We now added to Methods:

"Harmony was run with the default options, and the top 25 corrected Harmony PCs were selected to generate UMAP plots based on the ElbowPlot function in Seurat. Clustering analysis was performed on Harmony PCs via the *FindNeighbors* and *FindClusters* Seurat functions."

Reviewer 2

Faced with the inability to distinguish between spliced and unspliced mRNAs in the current single-cell sequencing analysis, leading to the possibility of misclassification of biological mechanisms in the final analysis results, the authors proposed a new SANSARA (Splicing-Aware scrNa-Seq AppRoAch) paradigm for analyzing the characterize peripheral blood regulatory T cell (Treg) subsets, revealing the interplay between FoxP3 and Helios master transcription factors and other unexpected splicing-informed features, with some innovative new.

Thank you for the understanding and positive view. Not only Tregs - the concept is general, we just applied it to helper T cells (since this is our field and we understand what is what), and discovered most interesting findings in Tregs.

1. The authors downloaded the raw data from the database, please inform the readers about the number of exons and introns to ensure the quality of the raw data and to guarantee the reproducibility, and also, what kind of pre-processing process is available for the raw data before performing the calculations;

Thank you for your suggestion.

We used standard FASTQC quality control to find low quality reads and assess adapter content.

We now added corresponding information to the Methods section.

We next used velocyto algorithm to calculate the number of spliced and unspliced UMI counts per cell and per gene (for highly expressed and variable genes used downstream for dimensionality reduction). All UMI designated as “spliced” contain no introns.

We now added **Supplementary Figure 1** which describes data at this level of analysis.

2. The authors used some software packages to perform the data analysis, and set the value of RESOLUTION during the analysis, with no other detailed parameters depicted, if there are any detailed parameter settings, please inform readers in the methodology;

SANSARA is generally run on Default function mode for different software and the result is quite stable on reasonable parameter settings. We added/modified the following information to the Methods section:

“Using the veloVI (v.0.3.0) package [10], we selected highly variable genes (by default – top-2000) and genes with a sufficient number of unspliced and spliced forms for further analysis. The sufficient number was evaluated by the veloVI function ‘`preprocess_data`’ by filtering out genes based on linear regression fit and on velocity fit. If velocity ‘gamma’ coefficient or linear regression coefficient were equal to zero, the gene was discarded from further analysis as poorly detected. For the selected genes, phase portraits reflecting the balance of spliced and unspliced forms were constructed. Out of 2000 highly variable genes, 253, 476 and 476 were selected in three donors for downstream analysis.

The splicing score was calculated for each gene in each cell based on the gene-specific phase portrait using veloVI framework and is basically a velocity value. The normalized expression of variable genes (calculated on conventional cellranger counts via *logNormalise* Seurat function with default parameters) was then multiplied by the splicing score value of each gene in each cell. We divided the resulting metric into spliced and unspliced—negative values were defined as the “expression” of the spliced form of the gene, while positive values described the unspliced form of the gene—and took the modulo values.

The resulting splicing-aware gene expression (saGEX) matrix of spliced/unspliced counts was used for downstream Seurat (v.5.0.1) normalization, dimensional reduction and clustering [68]. All TCR genes were excluded from the variable features used in dimensionality reduction and integration to avoid spurious clusters."

"The Harmony pipeline was used for the integration separately for the GEX and saGEX datasets of three donors (3050, 9430 and 9104 cells) [12]. These datasets were independently normalized using the *LogNormalise* function with default parameters and integration features (n=2997 for splicing-unaware datasets, n = 218 for SANSARA datasets) were selected with the *SelectIntegrationFeatures* function in Seurat. After merging the datasets, variable features of the merged object were set to selected integrated features. Principal Components (PCs) (n=50) were calculated from scaled integration features. Harmony was run with the default options, and the top 25 corrected Harmony PCs were selected to generate UMAP plots based on the ElbowPlot function in Seurat. Clustering analysis was performed on Harmony PCs via the *FindNeighbors* and *FindClusters* Seurat functions. Under a reasonable number of dimensions (15-30), the results were largely stable (data not shown). The integrated dataset contained 21584 cells. For comparing clustering between methods, several metrics were used. To compute silhouette scores we used cluster R package (v 2.1.2) at five resolution levels from 0 to 2.5, which corresponds to an increasing number of clusters [69]. Clustering trees were built using the 'clustree' R package for the same set of resolutions from 0 to 2.5 [70]. Trustworthiness and continuity metric were calculated for UMAP/Harmony corrected PCA of SANSARA and conventional analysis by the formulas described in sci-kit learn (sklearn's trustworthiness) and pyDRmetrics python toolkits [71, 72]. Jaccard index of similarity and Adjusted Rand Index were calculated by *linkClustersMatrix* and *pairwiseRand* function of bluster R package *(v 1.4.0)* on SANSARA and conventional cluster annotations [73]."

3. The authors analyzed peripheral blood regulatory T cell (Treg) subsets and found the direct discrepancy between spliced and unspliced RNA, can you annotate what biological mechanisms of misclassification will occur through Cellmarkers, to realize the transition from phenomenon to function;

Thank you for pushing us in the right direction.

We investigated deeper the Treg-relevant biology revealed with a splicing-aware approach, resulting in several highly relevant findings. We summarized this analysis in the Results section, including new **Table 1**:

"In *effector* T_{reg}s, splicing-aware differential gene expression analysis performed for the for the *naïve*, *activated* and *effector* T_{reg} clusters identified 58 upregulated spliced genes versus 70 genes revealed by the splicing-unaware approach, with only 14 genes overlapping (threshold log2FC > 1.5, **Supplementary Table 2, Table 1**).

Both approaches indicated upregulation of MHC-II machinery (*HLA-DR/DM/DQ*), consistent with enhanced antigen-specific suppressive capacity of HLA-DR^{+} T_{reg}s [35]. Both also highlighted CD39 (*ENTPD1* gene), an ectoenzyme that generates adenosine mediating A2A-dependent immunosuppression [36-38] and supports FOXP3^{+} T_{reg} stability [39], as well as *DUSP4* and *TRIB1*, potentially counter-balancing *effector* T_{reg} activity and proliferation [21, 40, 41].

Additionally, the splicing-aware analysis captured spliced form of *CD38* (contributes to adenosine-mediated immunosuppression [42], reported as a marker of highly immunosuppressive T_{reg}s [43],

ITGAL (LFA-1, strong T_{reg} -dendritic cells adhesion, critical for T_{reg} homeostasis [44, 45], *LEF1* (FOXP3-cooperating transcription factor stabilizing the T_{reg} program [46], and *PRF1* (perforin-dependent regulatory cytotoxicity [47, 48] (**Table 1**). Together, the spliced gene set was clearly more enriched for T_{reg}s effector functionality modules.

and in Discussion:

"Differential expression analysis performed for the naïve, activated and effector Treg clusters using splicing aware and splicing unaware approaches further revealed distinct gene sets enriched in *effector* T_{reg}s (**Supplementary Table 2, Table 1**).

Both splicing-aware and splicing-unaware analyses indicated upregulation of the MHC-II presentation machinery - including *HLA-DR*, *HLA-DM*, and *HLA-DQ* genes - required for enhanced antigen-specific suppressive function of effector T_{reg}s [35]. Both analyses revealed enrichment of CD39 (*ENTPD1* gene), an ectoenzyme that hydrolyzes extracellular ATP/ADP to AMP, which next mediates downstream immunosuppression via A2A receptors on effector T cells and antigen-presenting cells [36-38], and stabilizes FOXP3^{+} Tregs [39]. Both analyses also demonstrated increased expression of *DUSP4* and *TRIB1*, two genes potentially counterbalancing *effector* T_{reg}s functionality. DUSP4 dephosphorylates STAT5 and promotes its turnover, thereby limiting FOXP3-stabilizing STAT5 activity [21]. TRIB1 is a binding partner for FOXP3, which overexpression was associated with a decrease in Treg proliferative capacity [40, 41].

Furthermore, the splicing-aware approach identified several important genes involved in T_{reg} functionality (**Table 1**). In particular, SANSARA revealed that *effector* T_{reg}s express the spliced form of *CD38*, a marker previously associated with highly immunosuppressive T_{reg}s [43]. CD38 is an ectoenzyme that uses extracellular NAD^{+} to produce ADP ribose (ADPR), which can be further converted to AMP [42]. Therefore, CD39 and CD38 contribute complementary ecto-enzymatic cascades that convert extracellular ATP and NAD^{+} into adenosine, amplifying A2A-mediated immunosuppression when co-expressed on Tregs.

SANASARA also revealed that *effector* Tregs express spliced form of *ITGAL* (LFA-1, mediates strong Treg-dendritic cells adhesion, crucial role in T_{reg} function and homeostasis [44]), *PRF1* (mediates perforin-dependent T_{reg} cytotoxicity [47, 48]), *LEF1* (encodes a transcription factor that cooperates with FOXP3 to stabilize the T_{reg} program [46]).

Altogether, the spliced gene set was more enriched for effector T_{reg} functionality, indicating execution-state mRNA readiness. These findings have significant implications for our understanding of T_{reg} biology [65] and T_{reg}-based therapy developments [66], and clearly demonstrate SANSARA's ability to reveal biologically relevant mechanisms that remain hidden to splicing-unaware analyses."

4. Suggest whether the authors can use the expression matrices of spliced and unspliced mRNAs as independent modal inputs, and use multi-view dimensionality reduction methods (e.g., MOFA+, totalVI) to conduct joint analysis, because the results obtained by different algorithms may also have deviations.

Thank you for the suggestion. We have performed multimodal integration with Weighted Nearest Neighbor (WNN) analysis (https://doi.org/10.1016/j.cell.2021.04.048). Specifically, we split the dataset to spliced and unspliced saGEX gene forms. Then we separately postprocessed and integrated them (across donors) via Harmony as described in the paper. Thus, we obtained two Harmony-corrected PCAs, representing information from spliced and unspliced gene modalities. WNN pipeline (https://satijalab.org/seurat/articles/weighted_nearest_neighbor_analysis) was then used to learn cell-specific weight of each modality, and integrate them into WNN graph, as one would do with RNA-

seq/CITE-seq data. First 15 Harmony PCs from each modality were used as input to find multimodal neighbors and build UMAP and clustering.

The result closely resembled the pipeline with single PCA described in the paper, with minimal cluster distortions, as can be seen from the projection of annotation and Jaccard cluster similarity index (multimodal clustering produced several . The spliced/unspliced gene expression patterns are also the same, data shown for FOXP3, IKZF2 and IL10RA (**Response Figure 2**). Thus, multimodal dimensionality reduction did not deviate from the unimodal approach.

A.

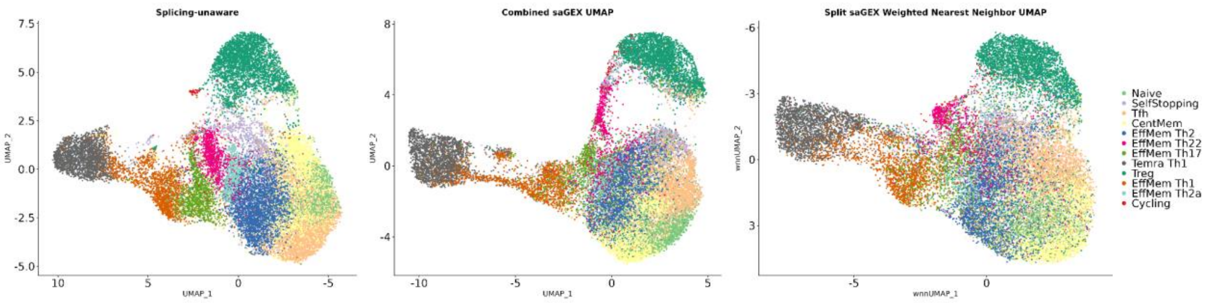

B.

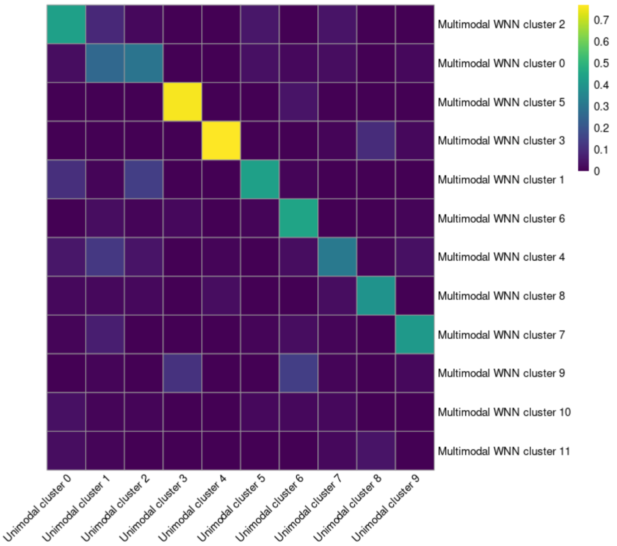

C.

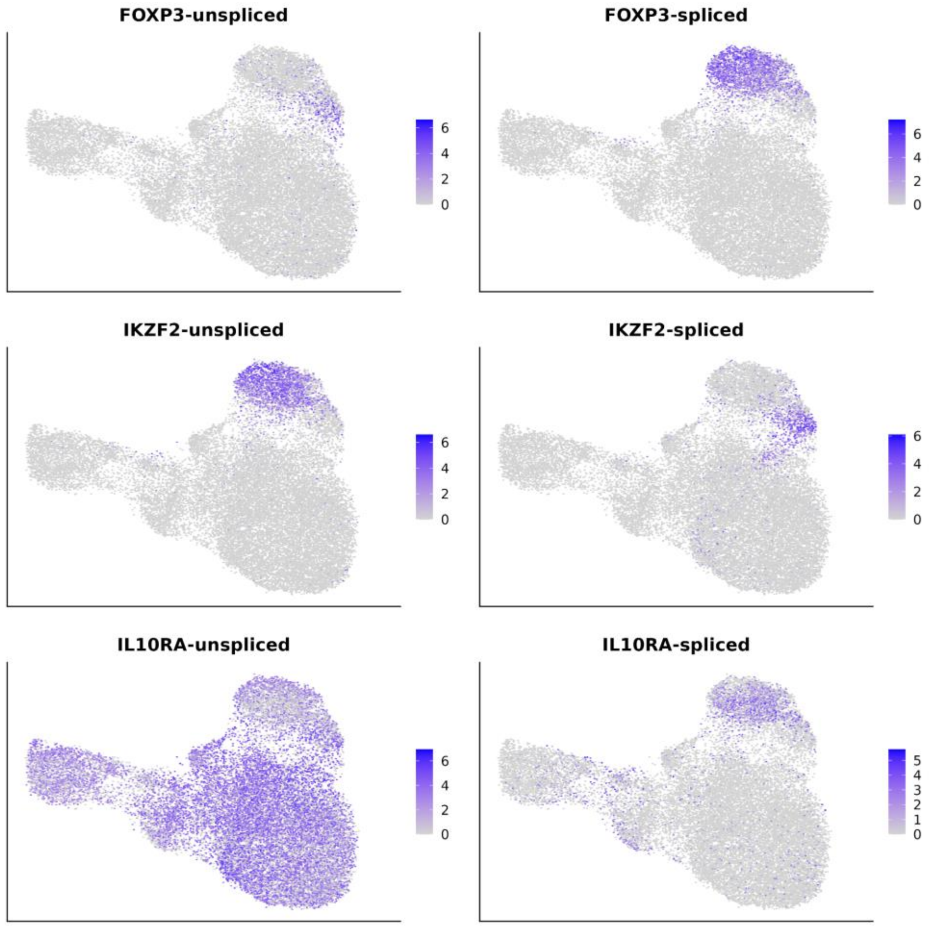

Response Figure 2. A. UMAP projection colored by the annotation of the a. Splicing-unaware dataset b. Unimodal splicing-aware dataset c. Multimodal splicing-aware dataset, B. Jaccard similarity index between clusters of Unimodal and Multimodal integration at resolution 0.7, C. Feature plots of saGEX gene expression (FOXP3, IKZF2, IL10RA) for Multimodal integration.

Reviewer 3

In their paper, the authors describe SANSARA (Splicing-Aware scrNa-Seq AppRoAch), a method that generates a splicing-adjusted gene expression matrix (saGEX) that estimates the amount of splicing for each gene in each cell. The resulting saGEX is then subjected to a conventional clustering and dimensionality reduction pipeline to reconstruct a representation of splicing that leads to the scRNA-Seq data.

This is a very promising approach that can be used by many immunologists in the field.

Thank you so much for the deep analysis and the positive view.

They applied this technique to characterise the splicing of human peripheral blood helper T cells, specifically regulatory T cell (Treg) subsets. This has revealed the crosstalk between the master transcription factors FoxP3 and Helios, Galectin3 - which plays a major role in regulating the cell fate of regulatory cells - and many other important features of the splisosome of Tregs. However, there are several points that require further attention. In particular, a more detailed description at several positions would really help to understand the study in more detail.

At this stage, I have several major comments:

1) In Figure 3, the authors take the top three hits, FOXP3, Helios and IL-10R. They then go on to describe hit number 6, which is galectin. In my opinion, it would be important to also show the UMAP profiles for DUSP4 and CARD16, which are also very important regulators of T cells, and to devote some attention to them in the manuscript.

We now expanded the discussion of relevant effector Treg genes identified with a splicing-aware approach - see new blocks in Results and Discussion sections, and a new **Table 1**.

DUSP4 and CARD16 UMAPs were added to the **Supplementary Fig. 6.**

2) The discussion is very short and should be expanded. The authors pretty much repeat what they wrote in their abstract: "… in Tregs, we uncovered reciprocal splicing interplay between the master transcription factors FoxP3 and Helios, alongside exclusive expression of the spliced form of IL10RA in activated and effector Tregs. These findings have significant implications for our understanding of Treg biology and Treg-based therapy developments. .. However, it would be important to expand which exactly implications it might have. Furthermore, they do not mention the other hits in the discussion like galectin3, which would be beneficial.

Thank you for the very right point. We worked further and now significantly expanded both Results and Discussion on Treg biology.

3) Materials and methods: the description is rather short and all chapters need to be expanded with more concrete information:

For example, the authors write:

..Using the veloVI (v.0.3.0) package10, we selected highly variable genes and genes with a sufficient number of unspliced and spliced forms for further analysis. ..
The Questions: How many genes were selected, what was the threshold value for the number of the spliced forms?

…. The normalized expression of variable genes was then multiplied by the splicing score value of each gene in each cell..

The question is how the splicing score was calculated?

We have expanded the Methods section and included detailed parameter settings. Specifically for the veloVI gene selection process:

"Using the veloVI (v.0.3.0) package [10], we selected highly variable genes (by default – top-2000) and genes with a sufficient number of unspliced and spliced forms for further analysis. The sufficient number was evaluated by the veloVI function 'preprocess_data' by filtering out genes based on linear regression fit and on velocity fit. If velocity 'gamma' coefficient or linear regression coefficient were

equal to zero, the gene was discarded from further analysis as poorly detected. For the selected genes, phase portraits reflecting the balance of spliced and unspliced forms were constructed. Out of 2000 highly variable genes, 253, 476 and 476 were selected in three donors for downstream analysis.

The splicing score was calculated for each gene in each cell based on the gene-specific phase portrait using veloVI framework and is basically a velocity value. The normalized expression of variable genes (calculated on conventional cellranger counts via *logNormalise* Seurat function with default parameters) was then multiplied by the splicing score value of each gene in each cell. “

Journal Requirements:

1) Please provide an Author Summary. This should appear in your manuscript between the Abstract (if applicable) and the Introduction, and should be 150-200 words long. The aim should be to make your findings accessible to a wide audience that includes both scientists and non-scientists. Sample summaries can be found on our website under Submission Guidelines: https://journals.plos.org/ploscompbiol/s/submission-guidelines#loc-parts-of-a-submission

Author Summary added.

2) Please upload all main figures as separate Figure files in .tif or .eps format. For more information about how to convert and format your figure files please see our guidelines: https://journals.plos.org/ploscompbiol/s/figures

Done.

3) We have noticed that you have uploaded Supporting Information files, but you have not included a list of legends. Please add a full list of legends for your Supporting Information files after the references list.

Done.

4) We notice that your supplementary Figures are included in the manuscript file. Please remove them and upload them with the file type 'Supporting Information'. Please ensure that each Supporting Information file has a legend listed in the manuscript after the references list.

Done.
