# Supplementary material for: Splicing-aware scRNA-Seq resolution reveals execution-ready programs in effector Tregs
Source: PLoS Comput Biol. 2025 Nov 10;21(11):e1013682. doi: 10.1371/journal.pcbi.1013682 (PMC12646427; doi:10.1371/journal.pcbi.1013682)
Supplement: Attachment — Submitted filename: reviewer .docx [file pcbi.1013682.s013.docx]

Faced with the inability to distinguish between spliced and unspliced mRNAs in the current single-cell sequencing analysis, leading to the possibility of misclassification of biological mechanisms in the final analysis results, the authors proposed a new SANSARA (Splicing-Aware scrNa-Seq AppRoAch) paradigm for analyzing the characterize peripheral blood regulatory T cell (Treg) subsets, revealing the interplay between FoxP3 and Helios master transcription factors and other unexpected splicing-informed features, with some innovative new.

1. The authors downloaded the raw data from the database, please inform the readers about the number of exons and introns to ensure the quality of the raw data and to guarantee the reproducibility, and also, what kind of pre-processing process is available for the raw data before performing the calculations;

2. The authors used some software packages to perform the data analysis, and set the value of RESOLUTION during the analysis, with no other detailed parameters depicted, if there are any detailed parameter settings, please inform readers in the methodology;

3. The authors analyzed peripheral blood regulatory T cell (Treg) subsets and found the direct discrepancy between spliced and unspliced RNA, can you annotate what biological mechanisms of misclassification will occur through Cellmarkers, to realize the transition from phenomenon to function；

4. Suggest whether the authors can use the expression matrices of spliced and unspliced mRNAs as independent modal inputs, and use multi-view dimensionality reduction methods (e.g., MOFA+, totalVI) to conduct joint analysis, because the results obtained by different algorithms may also have deviations.
